# Prevalence, co-infection and seasonality of fecal enteropathogens from diarrheic cats in the Republic of Korea (2016–2019): a retrospective study

**DOI:** 10.1186/s12917-021-03075-6

**Published:** 2021-12-01

**Authors:** Ye-In Oh, Kyoung-Won Seo, Do-Hyung Kim, Doo-Sung Cheon

**Affiliations:** 1 Laboratory of Veterinary Internal Medicine, College of Veterinary Medicine, Seoul National University, Seoul, 08826 Republic of Korea; 2 Knotus Inc., Incheon, 22014 Republic of Korea; 3 Postbio Inc., Guri-si, 11906 Republic of Korea

**Keywords:** Diarrhea, Feline, Infection, Real-time PCR

## Abstract

**Background:**

Diarrhea is one of the most common clinical symptoms in cats and can be caused by infectious pathogens and investigation of the prevalence, co-infection and seasonality of enteropathogens are not well-established in diarrheic cats.

**Results:**

Fecal samples of 1620 diarrheic cats were collected and enteropathogens were detected using real-time PCR. We retrospectively investigated the clinical features, total/seasonal prevalence, and infection patterns of enteropathogens. The positive infection rate was 82.59%. Bacterial, viral, and protozoal infections accounted for 49.3, 37.57, and 13.13% of cases, respectively. Feline enteric coronavirus (FECV) was the most common pathogen (29.37%), followed by *Clostridium* (*C.*) *perfringens*, *Campylobacter* (*C.*) *coli*, feline parvovirus, and *Tritrichomonas foetus*. The seasonality of enteropathogens was observed with peaks as follows: bacterial infections peaked in October, viral infections peaked in November, and protozoal infections peaked in August. Viral and protozoal infections showed differences in prevalence according to patient age. In the infection patterns, the ratios of single infections, mixed infections, and co-infections were 35.72, 9.87, and 54.41%, respectively. FECV was predominant in single infections. The most common patterns of multiple infections were *C. perfringens* and *C. coli* in mixed infections and *C. perfringens* and FECV in co-infections.

**Conclusions:**

Infection patterns differed according to the enteropathogen species, seasonality, and age distribution in cats. The results of this study might be helpful to understand in clinical characteristics of feline infectious diarrhea. In addition, continued monitoring of feline enteropathogens is required.

## Introduction

Diarrhea is the most common clinical symptom of feline gastrointestinal diseases [1, 2]. One of the common causes of diarrhea in cats is an infection, including various bacterial, viral, protozoal, and parasitic infections [1, 3, 4]. Many pathogens have been reported to present as mixed infections, co-infections, and single infections in cats. Only one pathogen can cause diarrhea; however, several pathogens can infect a single cat simultaneously owing to shared pathogenesis with a particular pathogen or a symbiotic relationship [5]. In these cases, because of the variability of enteropathogens, treatment of patients with multiple infections is more challenging than the treatment of those with single infections, resulting in increased economic costs. Studies of infection patterns can help to determine a patient’s treatment and prognosis. However, studies on the patterns of infection in cats with diarrhea are still limited [5–9].

Although the species and prevalence of some enteropathogens in diarrheic cats have been established, only a few studies have evaluated the relationships between specific pathogens and patient demographics or seasonality [1, 3–5, 10–12]. In humans, the relationships between various enteropathogens and other factors, such as etiology, seasonality, and age, have been studied extensively [13–17]. In addition, most feline enteropathogens have zoonotic potential, putting owners and veterinary staff at risk [11–18]. Further studies on enteropathogens in cats are needed [19–21]. Accordingly, in this study, we aimed to investigate the prevalence and infection patterns of enteropathogens in cats with diarrhea in the Republic of Korea. Additionally, we evaluated the clinical features of infections in cats, including seasonality and association with age.

## Materials and methods

### Data collection

A retrospective study was conducted on the results of the feline diarrheal real-time PCR panel test submitted by 145 animal hospitals for 37 months from April 1, 2016, to April 30, 2019, to a commercial veterinary diagnostic laboratory (PobaniLab, Republic of Korea). All cats were patients with acute or chronic diarrhea. Clinical information, including age, breed, and sex, was obtained from animal hospitals.

### Sample collection and transportation

At the animal hospitals, approximately 3–5 g of stool was obtained directly from the rectum or excreted feces and then placed into a sterile specimen cup. After collection, the stool samples were placed in a transport medium with a preservative and transported using a cooler bag (4–10 °C) to the laboratory within 2 h. All samples were tested within 2 h of arrival at the PobaniLab Veterinary Diagnostic Laboratory.

### Molecular detection of feline enteropathogens

#### Clinical samples

Stool specimens were collected from cats with gastroenteritis and shipped as stool suspension in universal transport media under refrigerated conditions. For real-time PCR, 150 μl of stool suspension was used for nucleic acid purification.

#### Nucleic acid purification and real-time PCR

Nucleic acids were extracted from the samples using the total nucleic acid purification kit (POSTBIO, Guri, Korea) based on the QIAcube platform (Qiagen) using a protocol tailored after the user’s extraction protocol with the best optimized conditions. For real-time PCR, 5 μL of nucleic acid was mixed with 20 μL of master mix from Qiagen for individual targets, and qPCR or qRT-PCR was performed using an Agilent AriaMx (Agilent, Santa Clara, CA, USA). All molecular assays were performed according to the standard laboratory instructions of PobaniLab. Based on previous studies, a C_T_ value of 40 or less was considered positive because there no specific C_T_ values have been shown to be positive for all pathogens.

#### Target gene and analytical sensitivity of qPCR or qRT-PCR

Target genes for enteropathogen detection using real-time PCR included genes from *Clostridium perfringens* (α-toxin gene, *cpa*) [22], *Campylobacter* (*C.*) *coli* (*gyrB*), *C. jejuni* (*rimM*) [23], *Salmonella* spp. (*invE*) [24], enterotoxigenic *Escherichia* (*E.*) *coli* (ETEC) (*ST* and *LT*), enteropathogenic *E. coli* (EPEC) (*bfpA*), enteroinvasive *E. coli* (EIEC) (*ipaH*), enterohemorrhagic *E. coli* (EHEC) (*stx1* and *stx2*) [25], feline enteric coronavirus (FECV) (membrane protein) [26], feline parvovirus (FPV) (*VP2*) [27], group A rotavirus (*nsp4*) [25], feline immunodeficiency virus (FIV) (*gag*) [28], *Tritrichomonas* (*T.*) *foetus* (*ITS-1*) [29], *Toxocara cati* (*ITS-1*) [30], *Toxoplasma gondii* (*RE*) [31], *Cryptosporidium parvum* (18 s rRNA), *Giardia* (*G.*) *lamblia* (18 s rRNA), *Entamoeba histolytica* (18 s rRNA) [25], and *Cyclospora* (*C.*) *cayetanensis* (18 s rRNA) [32]. Because the pathogenicity of *Escherichia* (*E.*) *coli* differs according to the subtype [18], we tested for ETEC, EPEC, EIEC, and EHEC (Table 1).

**Table 1:** The details of real-time PCR for the detection of feline enteropathogens

Pathogen	Target gene	Real-time PCR conditions		
		PCR protocol	Primer/Probe concentration	LOD^c^ (Based on Ct 40)
Feline coronavirus	M	RT PCR^a^	Primer: 10pmole/Rx Probe: 5pmole/Rx	10 copies
*C. perfringens*	cpa	PCR^b^	//	100 copies/Rx
*C. coli*	gyrB	PCR	//	100 copies/Rx
Feline parvovirus	vp2	PCR	//	10 copies/Rx
*T. foetus*	ITS1	PCR	//	100 copies/Rx
EPEC	bfpA	PCR	//	100 copies/Rx
*Giardia lamblia*	18 s rRNA	PCR	//	10 copies/Rx
ETEC	ST/LT	PCR	//	100 copies/Rx
*C. jejuni*	rimB	PCR	//	100 copies/Rx
EHEC	stx1/stx2	PCR	//	10 copies/Rx
Group A Rotavirus	nsp4	RT PCR	//	10 copies/Rx
*C. parvum*	18 s rRNA	PCR	//	100 copies/Rx
*C. cayetanensis*	18 s rRNA	PCR	//	100 copies/Rx
EIEC	ipaH	PCR	//	100 copies/Rx
*Salmonella* spp.	invE	PCR	//	100 copies/Rx
*E. histolytica*	18 s rRNA	PCR	//	100 copies/Rx
*Toxocara cati*	ITS1	PCR	//	100 copies/Rx
*Toxoplasma gondii*	RE	PCR	//	100 copies/Rx
FIV	gag	PCR	//	100 copies/Rx

*C. perfringens Clostridium perfringens*, *C. coli Campylobacter coli*, *T. foetus Tritrichomonas foetus*, *EPEC* enteropathogenic *Escherichia coli*, *ETEC* enterotoxigenic *Escherichia coli*, *C. jejuni Campylobacter jejuni*, *EHEC* enterohemorrhagic *Escherichia coli*, *C. parvum Cryptosporidium parvum*, *C. cayetanensis Cyclospora cayartensis*, *EIEC* enteroinvasive *Escherichia coli*, *E. histolytica Entamoeba histolytica*, *FIV* feline immunodeficiency virus

^a^PCR thermal condition: 95 °C, 5 min (95 °C, 10 s – 60 °C, 30 s; 45 cycles)

^b^RT PCR thermal condition: 50 °C, 15 min – 95 °C, 5 min (95 °C, 10 s – 60 °C, 30 s; 45 cycles)

^c^LOD (limitation of detection) was determined to be 10 folds serial dilutions of synthetic plasmid including target gene of individual pathogen based on a C_T_ value of 40

To determine the analytical sensitivity (the lower limit of detection) for individual target genes for enteric pathogens, serial diluents (10^5^ to 1 copies/reaction) of synthetic DNAs or transcript RNAs for enteric pathogens were analyzed using qPCR or qRT-PCR. The lower limit of detection was defined as the lowest concentration that was detected in ≥95% of the replicates [13].

### Data and statistical analyses

In this study, a mixed infection was defined as a case in which different substances from the same class, such as two types of bacterial species, were detected together, and a co-infection was indicated when different types of pathogens were detected together, e.g., bacteria and viruses, in the same fecal specimen [33].

Continuous variables, such as age, were presented as mean, minimum, and maximum values. Categorical variables were expressed as numbers and percentages. To compare the prevalence of enteropathogens according to feline age, Chi-square tests were performed. Cochran-Armitage tests for trends were used to assess the seasonality of the prevalence of enteropathogens. The threshold for statistical significance was set to a *P*-value of less than 0.05. Data were analyzed using a statistical computer software (Prism 6 Version 6.01; Graphpad).

## Results

### Clinical features of feline patients with diarrhea

We obtained fecal samples and clinical information from 1620 cats showing symptoms of diarrhea from 145 small animal hospitals in the Republic of Korea. The number of cats per animal hospital was a minimum of 1, a maximum of 171, and a median of 2.

There were 32 breeds of cats represented, with 693 mixed breed cats (42.8%) and 927 (57.2%) pure breed cats. The most common breeds were 664 domestic shorthair cats (41%), 100 Munchkins (6.2%), 100 Persians (6.2%), 96 Scottish folds (5.9%), 88 Siameses (5.4%), 83 Russian Blues (5.1%), 77 Abyssinians (4.8%), 65 Bengals (4%), 57 Ragdolls (3.5%), and 50 Norwegian Forests (3.1%).

In this study, 357 male cats, 430 castrated male cats, 342 female cats, 278 spayed female cats, and 213 cats of undocumented sex were included. Due to insufficient data on sex, we did not perform a sub-analysis of sex-specific prevalence. The mean age was 3.3 years (range, 4 months to 19 years). Cats were categorized into three groups based on age as follows: < 1 year old (early age group; *n* = 168), 1–7 years old (middle age group; *n* = 1267), and > 8 years old (old age group; *n* = 185). Among these cats, fecal real-time PCR tests of 82.59% (1338/1620) were positive for enteropathogens.

### Total prevalence of feline enteropathogens

FECV, *Clostridium perfringens*, *C. coli*, feline parvovirus (FPV), and *T. foetus* were predominant infections (Table 2). The rates of infection for bacteria, viruses, and protozoa were 49.3% (*n* = 1378), 37.57% (*n* = 1050), and 13.13% (*n* = 367), respectively. *E. coli* accounted for 8.66% of the total cases, with most cases being enteropathogenic *E. coli* (EPEC). The top-10 most frequently detected pathogens accounted for 96.67% (2702/2795) of all cases.

**Table 2:** Prevalence of enteropathogens from feline fecal specimens based on age

Enteropathogen	Total prevalence, % (n)	Age			
		< 1 year	1–7 years	≥ 8 years	*P*-value
Feline coronavirus	29.37 (819)	31.33 (104)	29 (643)	30 (72)	< 0.0001
*C. perfringens*	23.84 (665)	19.58 (65)	23.73 (526)	30.83 (74)	0.75
*C. coli*	14.88 (415)	17.17 (57)	15.25 (338)	8.3 (20)	< .0001
Feline parvovirus	7.49 (209)	5.72 (19)	7.76 (172)	7.5 (18)	0.28
*T. foetus*	6.7 (187)	7.23 (24)	6.9 (153)	4.17 (10)	0.015
EPEC	4.7 (131)	7.53 (25)	4.19 (93)	5.42 (13)	0.0029
*Giardia lamblia*	4.52 (126)	4.82 (16)	4.83 (107)	1.25 (3)	0.0036
ETEC	2.62 (73)	3.31 (11)	2.21 (49)	5.42 (13)	0.06
*C. jejuni*	1.76 (49)	2.11 (7)	1.71 (38)	1.67 (4)	0.54
EHEC	1 (28)	0 (0)	1.08 (24)	1.67 (4)	0.19
Group A Rotavirus	0.79 (22)	0 (0)	0.9 (20)	0.84 (2)	0.24
*C. parvum*	0.75 (21)	0.3 (1)	0.81 (18)	0.84 (2)	0.6
*C. cayetanensis*	0.68 (19)	0.3 (1)	0.72 (16)	0.84 (2)	0.75
EIEC	0.36 (10)	0 (0)	0.32 (7)	1.25 (3)	0.12
*Salmonella* spp.	0.25 (7)	0.3 (1)	0.27 (6)	0 (0)	0.62
*E. histolytica*	0.11 (3)	0 (0)	0.13 (3)	0 (0)	0.66
*Toxocara cati*	0.07 (2)	0.3 (1)	0.05 (1)	0 (0)	0.18
*Toxoplasma gondii*	0.07 (2)	0 (0)	0.09 (2)	0 (0)	0.76
FIV	0.04 (1)	0 (0)	0.05 (1)	0 (0)	0.88
Total	100 (2789)	100 (332)	100 (2217)	100 (240)	

*C. perfringens Clostridium perfringens*, *C. coli Campylobacter coli*, *T. foetus Tritrichomonas foetus*, *EPEC* enteropathogenic *Escherichia coli*, *ETEC* enterotoxigenic *Escherichia coli*, *C. jejuni Campylobacter jejuni*, *EHEC* enterohemorrhagic *Escherichia coli*, *C. parvum Cryptosporidium parvum*, *C. cayetanensis Cyclospora cayartensis*, *EIEC* enteroinvasive *Escherichia coli*, *E. histolytica Entamoeba histolytica*, *FIV* feline immunodeficiency virus

The prevalence of each enteropathogen between age groups was compared statistically using the Chi-square test. *P < 0.05

### Seasonal prevalence of feline enteropathogens

**Fig. 1:**
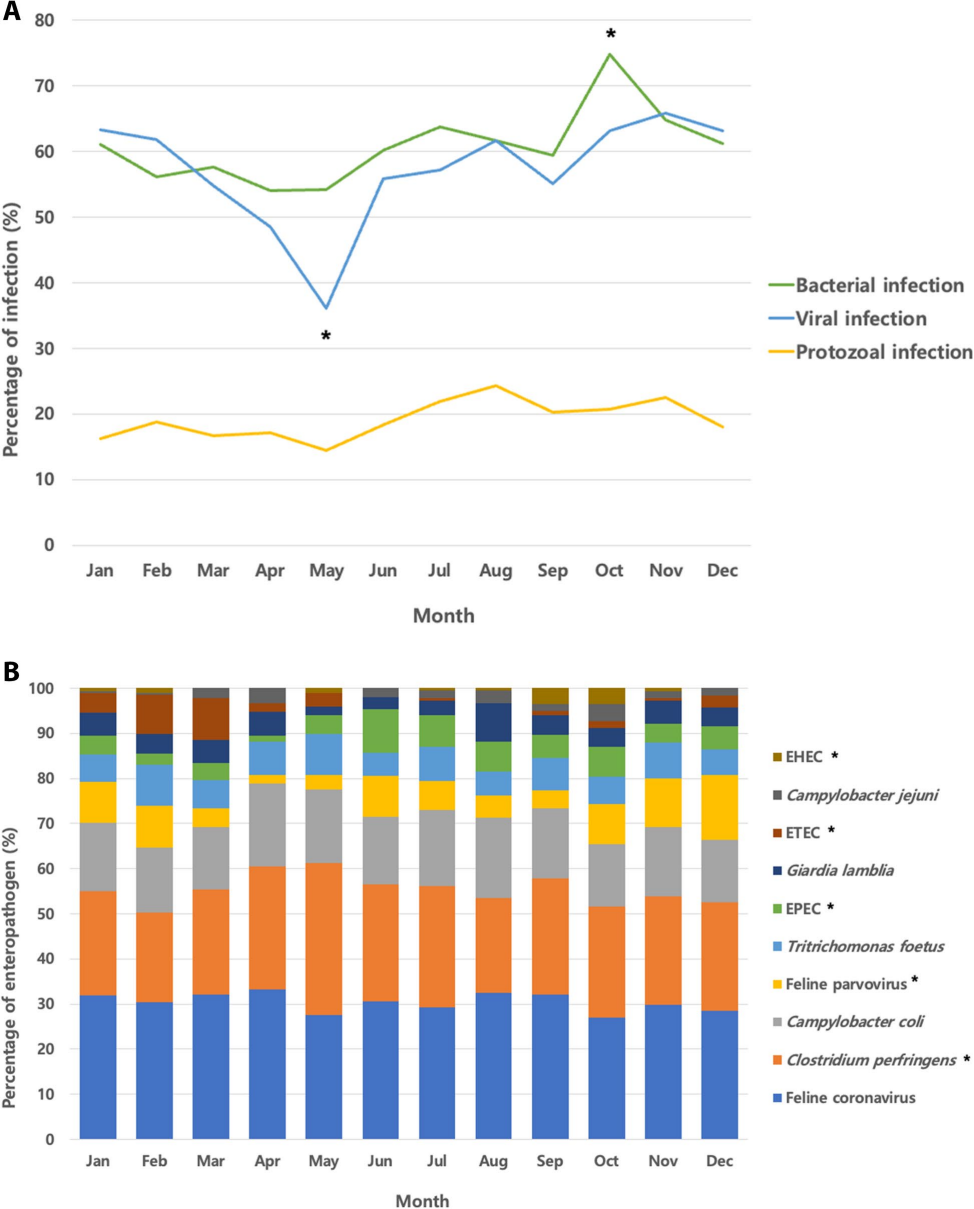
Seasonal distribution of enteropathogens from January to December in 1620 feline patients with diarrhea. **A** Prevalence of bacterial, viral, and protozoal infections by month in all feline patients. **B** Prevalence of the 10 most common enteropathogens in each month of this study. The infection rates of enteropathogens for each month were compared statistically using the Chi-square test for trend. *P < 0.05

There were differences in seasonal prevalence only in bacterial and viral infections (*P* = 0.01 and 0.04, respectively; Fig. 1A) but not in protozoal infections. Bacterial infections showed the lowest prevalence in April, while viral and protozoal infections showed the lowest prevalence in May. Bacterial infections showed a high prevalence in October, viral infections showed a high prevalence in November, and protozoal infections showed a high prevalence in August. FECV, *Clostridium perfringens*, and *C. coli* were detected at high rates throughout the year (Fig. 1B). The infection rates of *Clostridium perfringens*, FPV, EPEC, ETEC, and EHEC changed significantly according to the month.

### Association between feline enteropathogens and age

**Fig. 2:**
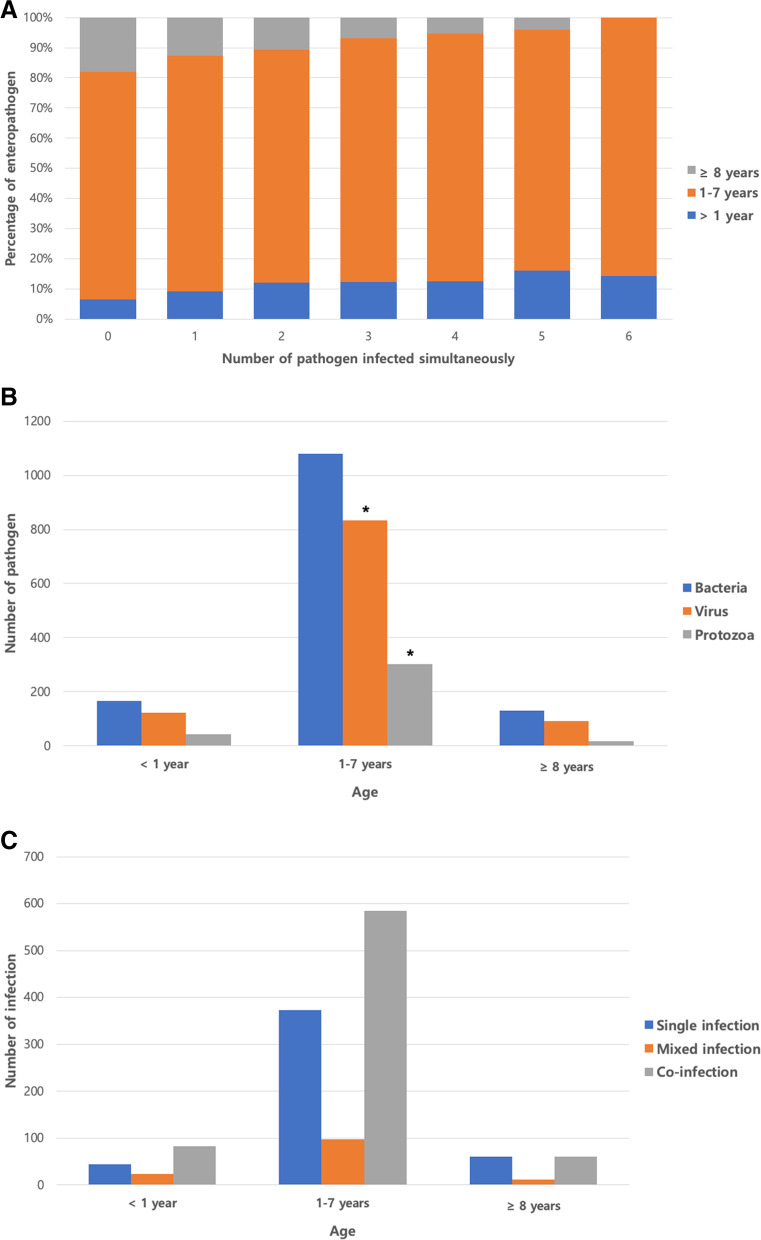
Frequency of detection of multiple enteropathogens in 1620 fecal specimens based on feline patient age. **A** Comparison of the number of enteropathogens simultaneously detected based on patient age. **B** Comparison of the number of bacterial, viral, and protozoal enteropathogens based on patient age. **C** Comparison of infection type based on patient age. The infection rates of enteropathogen between age groups were compared statistically using the Chi-square test. *P < 0.05

RT-PCR results showed that the percentages of positive and negative infections were 82.59% (1338) and 17.41% (282), respectively. Among positive infections, each individual was infected with 1–6 pathogens at the same time; single, double, triple, quadruple, quintuple, and sextuple infection rates were 29.51% (478), 27.96% (453), 16.17% (262), 6.98% (113), 1.54% (25), and 0.43% (7), respectively (Fig. 2A). The percentages of positive infections in the early age, intermediate age, and old age groups were 11.21% (150), 78.77% (1054), and 10.01% (134), respectively. Across all three age groups, bacterial infections were most common (Fig. 2B). The infection rates of viruses and protozoa were different according to age (*P* = 0.0006 and 0.0009, respectively).

### Association between feline enteropathogens and infection patterns

**Fig. 3:**
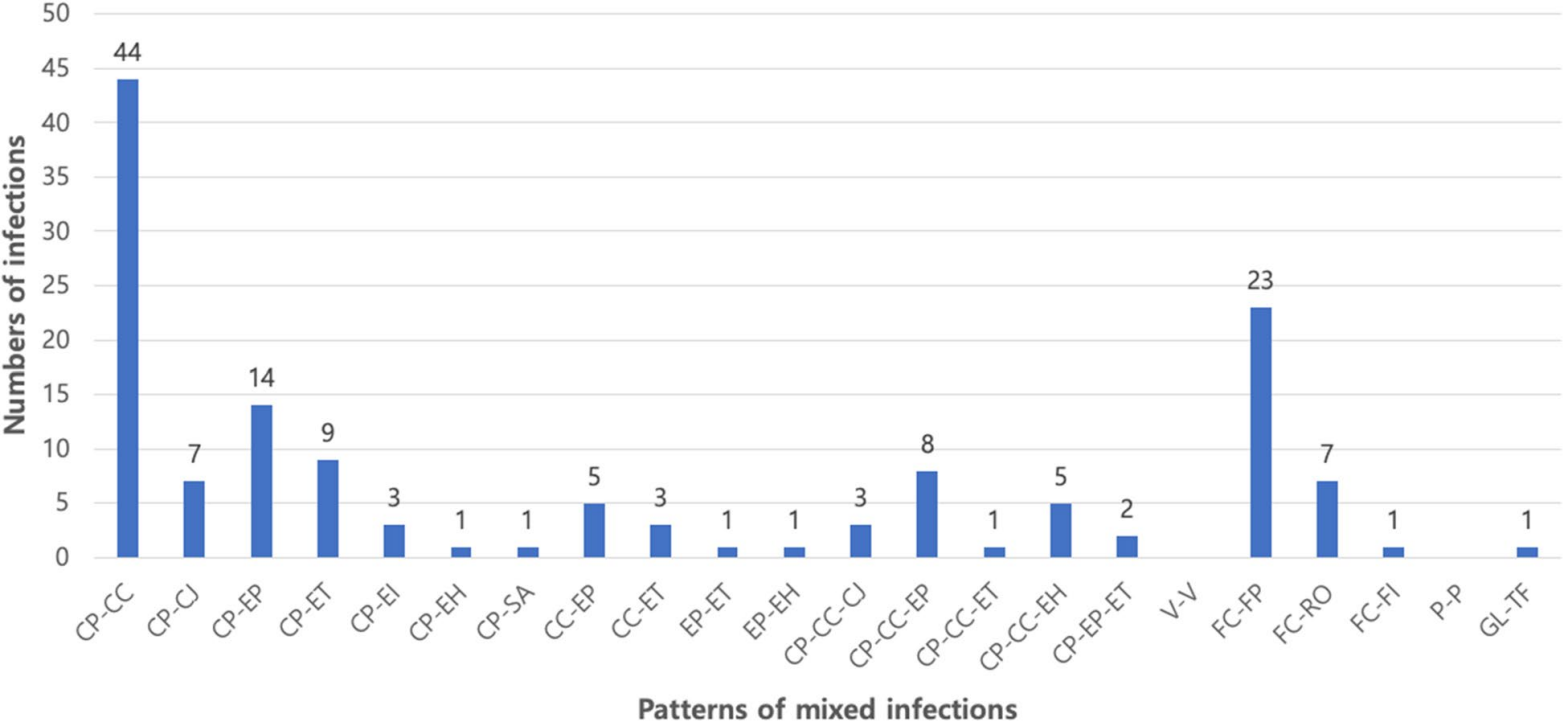
Frequency of mixed infection patterns of enteropathogens from fecal specimens in 1620 feline diarrheic patients. CC, *Campylobacter coli*; CJ, *Campylobacter jejuni*; CP, *Clostridium perfringens*; EH, enterohemorrhagic *Escherichia coli*; EI, enteroinvasive *Escherichia coli*; EP, enteropathogenic *Escherichia coli*; ET, enterotoxigenic *Escherichia coli*; FECV, Feline coronavirus; FI, Feline immunodeficiency virus; FP, Feline parvovirus; GL, *Giardia lamblia*; RO, Group A Rotavirus; SA, *Salmonella* spp.; TF, *Tritrichomonas fetus*

**Fig. 4:**
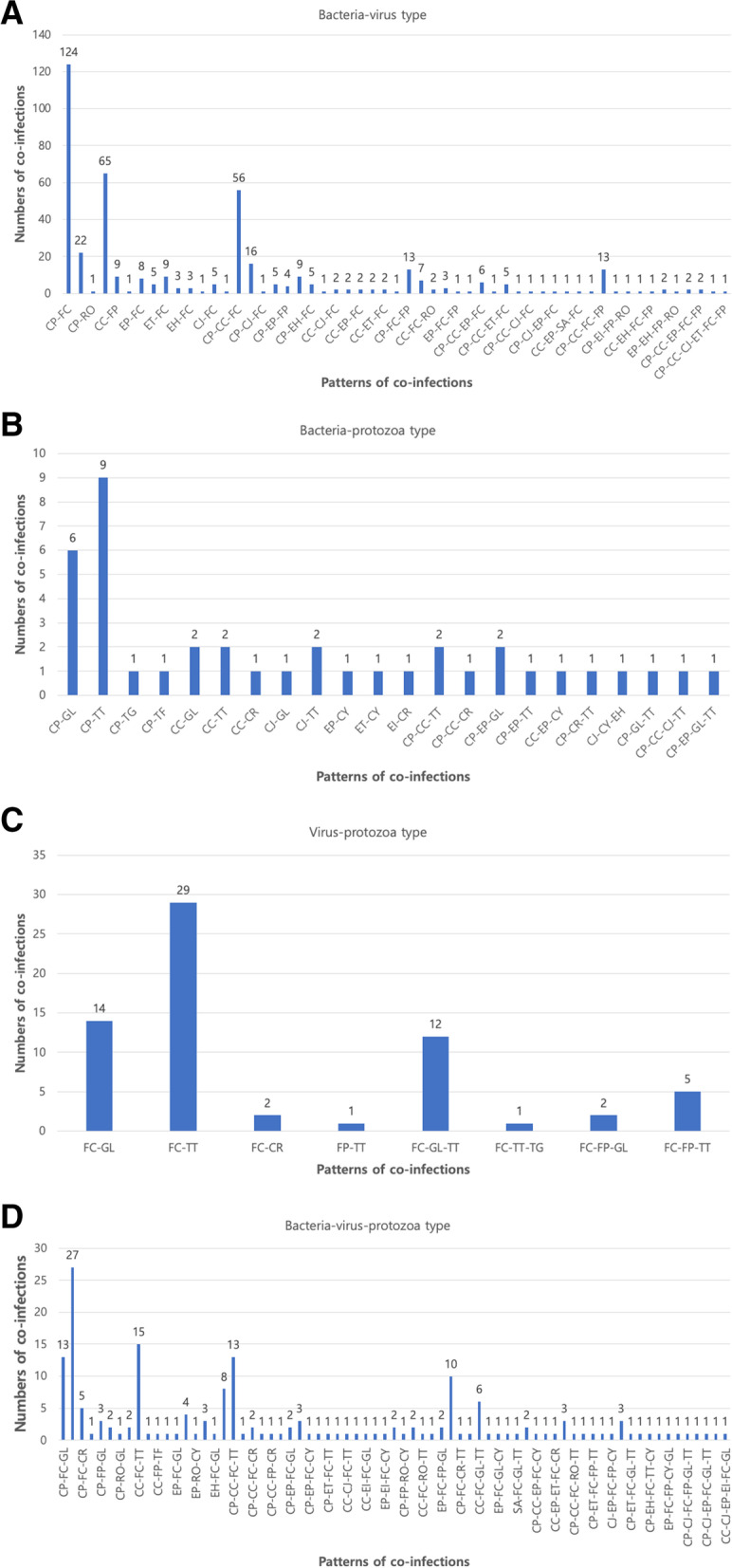
Frequency of co-infection patterns of enteropathogens from fecal specimens in 1620 feline diarrheic patients. **A** Type of bacterial-viral co-infection. **B** Type of bacterial-protozoal co-infection. **C** Type of viral-protozoal co-infection. **D** Type of bacterial-viral-protozoal co-infection. CC, *Campylobacter coli*; CJ, *Campylobacter jejuni*; CP, *Clostridium perfringen*; CR, *Cryptosporidium parvum*; CY, Cyclospora cayartensis; EH, enterohemorrhagic *Escherichia coli*; EI, enteroinvasive *Escherichia coli*; EP, enteropathogenic *Escherichia coli*, ET, enterotoxigenic *Escherichia coli*; FECV, Feline coronavirus; FI, Feline immunodeficiency virus; FP, Feline parvovirus; GL, *Giardia lamblia*; RO, Group A Rotavirus; SA, *Salmonella* spp.; TF, *Toxocara felis*; TG, Toxoplasma gondii; TT, *Tritrichomonas fetus*

Among the cases evaluated, 35.72% (478), 9.87% (132), and 54.41% (728) were single infections, mixed infections, and co-infections, respectively. Co-infection was the most common among all groups (Fig. 2C). Among single infections, 48.75% (233) were bacterial infections, 46.44% (222) were viral infections, and 4.81% (23) were protozoan infections (Table 3). Similar to the total prevalence, feline enteric coronavirus (FECV), *Clostridium perfringens*, and *C. coli* were the most common pathogens causing single infections. The infection patterns of mixed infections are shown in Fig. 3. Bacterial-bacterial infections accounted for 77.14% (108) of cases, whereas viral-viral and protozoal-protozoal infections accounted for 22.14% (31) and 0.71% (1) of the cases, respectively. Among mixed infections, the combination infection rate of *Clostridium perfringens* and *C. coli* was high. Co-infections included bacterial-viral (B-V) infections, bacterial-protozoal (B-P) infections, viral-protozoal (V-P) infections, and bacterial-viral-protozoal (B-V-P) infections. B-V infections accounted for the largest proportion (60.36%; 437). The next highest proportions were B-V-P infections (25%; 181), V-P infections (9.12%; 66), and B-P infections (5.52%; 40; Fig. 4A–D). The most common combinations in each group were *Clostridium perfringens* and FECV (28.38%) in the B-V pattern, *Clostridium perfringens* and *T. foetus* (22.5%) in the B-P pattern, FECV and *T. foetus* (43.94%) in the V-P pattern, and *Clostridium perfringens*, FECV, and *T. foetus* (14.92%) in the B-V-P pattern.

**Table 3:** Prevalence and infection patterns of enteropathogens from fecal specimens in cats with diarrhea

Pathogen	Prevalence of pathogen % (n)		
	Single infection	Mixed infection	Co-infection
Feline coronavirus	39.33 (188)	11.27 (31)	29.47 (600)
*C. perfringens*	26.78 (128)	32.73 (90)	21.95 (447)
*C. coli*	12.76 (61)	22.18 (61)	14.39 (293)
Feline parvovirus	7.11 (34)	8.36 (23)	7.47 (152)
*T. foetus*	2.3 (11)	0.36 (1)	8.6 (175)
EPEC	3.77 (18)	10.18 (28)	4.17 (85)
*Giardia lamblia*	1.46 (7)	0.37 (1)	5.8 (118)
ETEC	3.14 (15)	5.81 (16)	2.06 (42)
*C. jejuni*	1.26 (6)	3.27 (9)	1.67 (34)
EHEC	0.42 (2)	1.45 (4)	1.08 (22)
Group A Rotavirus	0 (0)	2.55 (7)	0.69 (14)
*C. parvum*	0.42 (2)	0 (0)	0.98 (20)
*C. cayetanensis*	0.42 (2)	0 (0)	0.83 (17)
EIEC	0.42 (2)	0.73 (2)	0.29 (6)
*Salmonella* spp.	0.21 (1)	0.37 (1)	0.25 (5)
*E. histolytica*	0.21 (1)	0 (0)	0.1 (2)
*Toxocara cati*	0 (0)	0 (0)	0.1 (2)
*Toxoplasma gondii*	0 (0)	0 (0)	0.1 (2)
FIV	0 (0)	0.37 (1)	0 (0)
Total	100 (478)	100 (275)	100 (2036)

*C. perfringens Clostridium perfringens*, *C. coli Campylobacter coli*, *T. foetus Tritrichomonas foetus*, *EPEC* enteropathogenic *Escherichia coli*, *ETEC* enterotoxigenic *Escherichia coli*, *C. jejuni Campylobacter jejuni*, *EHEC* enterohemorrhagic *Escherichia coli*, *C. parvum Cryptosporidium parvum*, *C. cayetanensis Cyclospora cayartensis*, *EIEC* enteroinvasive *Escherichia coli*, *E. histolytica Entamoeba histolytica*, *FIV* feline immunodeficiency virus

## Discussion

In this study, we evaluated the prevalence, clinical features, and seasonality of feline enteropathogens in the Republic of Korea. This is the first large-scale study to evaluate the clinical features and prevalence of 19 feline enteropathogens using real-time RT-PCR in the Republic of Korea. Most of the diarrheic cats in our study were diagnosed with enteropathogens, with FECV, *Clostridium perfringens*, *Campylobacter* spp., *E. coli*, and FPV being the more common pathogens isolated from the stool of diarrheic cats. These findings are similar to the results of previous studies [1, 3, 4, 11, 12], but differed somewhat from previous studies. The discrepancies could be related to a variety of factors, such as differences in methodologies, regions, living environments, and concurrent diseases, thereby affecting the species and prevalence of the identified enteropathogens. Bacterial infections were predominant, although the most common enteropathogen in this study was FECV. Notably, bacterial infections peaked in October, viral infections peaked in November, and protozoal infections peaked in August. Viral and protozoal infections showed differences in prevalence according to feline age. When the infection patterns were investigated, the ratios for single infections, mixed infections, and co-infections were 35.72, 9.87, and 54.41%, respectively. FECV was the predominant among single infections, and the most common infection patterns were a combination of *Clostridium perfringens* and *C. coli* in mixed infections and a combination of *Clostridium perfringens* and FECV in co-infections.

FECV was frequently identified in this study. Because FECV is ubiquitous and mostly subclinical, this study might have also had a high proportion of subclinical FECV infections [34]. In our study, however, the threshold cycle (C_T_) value was used to quantify the viral load to determine whether the infection was positive. FECV showed the highest prevalence among single infections, and the C_T_ value and viral load were correlated in real-time PCR [35]. Therefore, FECV is a major pathogen to consider for the diagnosis of infectious feline diarrhea.

*Clostridium perfringens* is a predominant microorganism in the feces of most carnivores [18, 36]. Because *Clostridium perfringens* is a normal inhabitant of the intestines of cats that do not have diarrhea [34], PCR analysis of *Clostridium perfringens* is difficult when attempting to determine whether this organism is the causative agent of diarrhea. α-Toxin expressed by *Clostridium perfringens* was detected in this study. Enterotoxins, rather than *Clostridium perfringens* itself, can cause enteritis, and our findings are based on the results of DNA amplification of α-toxin [18]. Previous studies have shown that α-toxin from *Clostridium perfringens* was only detected in fecal samples from diarrheic cats and not that from asymptomatic cats [12, 36]; however, opposing results have also been reported [1]. Thus, further studies are needed to determine whether *Clostridium perfringens* is a cause of diarrhea.

*Campylobacter* spp. is the main cause of infectious diarrhea in cats [37, 38], and it showed the third highest prevalence among all organisms in our study. In previous studies, *Campylobacter* spp. was detected in both diarrheic and healthy cats [39, 40], and the prevalence of *Campylobacter* spp. in diarrheic cats was higher than that in clinically healthy cats [41]. However, in some studies, similar rates have been detected in healthy cats [39, 40, 42], supporting the possibility of subclinical infection. Alternatively, these discrepancies may be related to differences in test methods, which include bacterial culture, enzyme-linked immunosorbent assay, and PCR. *Campylobacter helveticus*, *Campylobacter upsaliensis*, and *C. jejuni* are also frequently detected in cats [39]. Because *C. coli* and *C. jejuni* are the most common pathogens owing to their thermophilic characteristics [20], we evaluated these two pathogens as a PCR panel [38]. In contrast to previous studies in cats, dogs, and humans, in which *C. jejuni* was found to be a predominant bacterium, our results demonstrated that *C. jejuni* was less frequent than *C.coli* [41, 43, 44]. The prevalence of *Campylobacter* spp. may vary according to species, age, underlying disease, season, region, and concurrent infection, which could explain the higher rates of *C. coli* [20, 45]. However, because the results of this study were different from those of previous studies, further investigation on subspecies in feline campylobacteriosis is needed.

*E. coli* is a well-known colonic microorganism, and its pathogenicity depends on various virulence factors [18]. Among known diarrheagenic *E. coli* in cats, our study showed decreased prevalence in the order of EPEC > ETEC > EHEC > enteroinvasive *E. coli* (EIEC). When combined altogether, the prevalence of *E. coli* was the fourth most common. EPEC was the most abundant subtype among *E. coli* in our study, consistent with a previous report [46]. However, few studies have reported fecal detection of ETEC, EHEC, and EIEC in diarrheic cats, and EPEC and EHEC are known to also be present in healthy cats.

FPV is a pathogen that is included in the core vaccination schedule for cats [47]. In this study, we did not know the recent vaccine status of the enrolled cats; therefore, it is possible that false-positive results were obtained in individuals who had recently received modified live-virus vaccines [18]. In our study, because the prevalence of FPV was higher in the intermediate and old age groups than in the early age group, the possibility of subclinical infection due to young age was low.

*T. foetus* and *G. lamblia* were the most common protozoal infections in this study, similar to previous reports [48]. *T. foetus* infection is an emerging gastrointestinal disease in cats [49]. Although *T. foetus* is not a member of the microflora, this organism was detected in fecal samples in cats with or without diarrhea [50]. The pathogenesis of *T. foetus*-associated diarrhea in cats is not fully known [51]. To date, chronically infected cats have remained in clinical remission; however, diarrhea recurs because of food changes or stress [52]. Although subclinical infection is common for *G. lamblia*, clinical symptoms are also common because of its high infectivity, particularly in kittens and catteries. *G. lamblia* is a potential zoonotic protozoan that infects most mammals and causes diarrhea [34]. Although subclinical infections are often encountered, even if the test result is positive for *G. lamblia*, veterinarians should pay close attention to hygiene when handling these cats.

In our study, the feline immunodeficiency virus (FIV) was detected in only one cat. This cat was in the intermediate age group and presented as a mixed infection with FECV. It is possible that the intestinal microflora was infected by FIV. The relationship between FIV infection and various other enteropathogens has not yet been elucidated.

The seasonal distribution of the infection may vary according to the pathogen species. In our study, we analyzed the seasonality of the 10 most prevalent enteropathogens, as well as the seasonality of infection by species type: bacterial, viral, or protozoal. Bacterial and viral infections showed significant differences in prevalence according to the month, whereas protozoal infections did not. In veterinary medicine, few studies have evaluated seasonality by infection type [43, 53]. In our study, *Clostridium perfringens*, FPV, EPEC, ETEC, and EHEC showed significant differences in prevalence according to the time of year. Infections from FECV and *Campylobacter* spp., the most commonly identified pathogens in our study, occurred evenly throughout the year with no seasonality, as in previous studies [43]. In contrast, infection by *Campylobacter* spp. is more frequent in the summer and fall in cats [53]. However, it is difficult to conclude the clinical significance of these results. We only revealed the descriptive data of every month. Because there are few similar studies in veterinary medicine, making comparisons with the results of our study is difficult. Second, since this study was conducted locally, it is difficult to represent the infection in other regions. Third, these are the results of testing by only one diagnostic laboratory; hence, if we combine these with the test results of another diagnostic laboratory, other infection patterns may be observed. In a previous human study, climate, the biological properties of pathogens, and people’s eating habits could affect the seasonality of infectious diarrhea [13]. Additional long-term, large-scale studies of seasonality are needed in feline infectious diarrhea.

Some pathogens are more common in felines of certain ages [37, 49, 54]. In our study, there were differences in prevalence according to feline age for FECV, *C. coli*, *T. foetus*, and EPEC. EIEC, FECV, *C. coli*, and EPEC were more prevalent in the early age group. In kittens, the prevalence of pathogens is higher because kittens have lower antibody formation than adult cats [18, 50]. For example, FECV-associated diarrhea is most likely to occur in young kittens [18]. In our study, the prevalence of FECV (31.33%) was highest in cats under 1 year of age. Other studies have reported higher *Campylobacter* spp. prevalence rates in cats under 1 year of age [39, 41, 43]. In contrast, *C. jejuni* showed no differences in prevalence according to age, whereas *C. coli* differed among the age groups. EPEC also showed the highest prevalence in the early age group (7.53%), whereas EIEC and ETEC showed the highest prevalence in the old age group (1.25 and 5.42%, respectively). To date, studies on the relationships between the prevalence of enteropathogens and the age of cats are limited in cats with *E. coli*-associated diarrhea. However, in this study, the prevalence of *G. lamblia* was higher in the early age group (4.82%) and the intermediate age group (4.83%) than in the old age group (1.25%). *G. lamblia* and *T. foetus* had significantly higher prevalence rates in cats under 1 year of age than in older cats [5]. In contrast, in our study, the prevalence of *T. foetus* was not associated with age. Additionally, the proportion of *Clostridium perfringens* was significantly higher in the old age group, similar to a previous study demonstrating higher fecal concentrations of bacteria in cats over 9 years old [55]. However, because the number of cats in each age group was not balanced, this might affect the results of this study. The results should be interpreted in consideration of this point. Further study is needed to ensure that the number of cats in each age group is balanced.

The percentage of multiple infections in our study was 64.28%, which was higher than that in a previous study (44%) [1]. Concurrent infection with other pathogens can increase the severity of symptoms in some cats because of the possibility of sharing pathogenesis and symbiotic relationships [5, 18], e.g., co-infection with FPV and *Salmonella* spp. [56]. Fluctuations in the intestinal microbiota may cause second and third infections [57]. In this study, combination infection with *Clostridium perfringens* plus *C. coli* was predominant in mixed infections. Co-infections were also commonly detected as *Clostridium perfringens* plus FECV and *C. coli* plus FECV. Although the prevalence of protozoal infections was lower than that of bacterial and viral infections, such infections play important roles in infection patterns. In cats positive for *T. foetus*, an increased severity of diarrhea is associated with the presence of *Cryptosporidium* [58]. *Giardia* and *Cryptosporidium* are also interdependent [59]. Our findings are similar to previous findings demonstrating that co-infection-associated protozoa were primarily *Giardia* and *T. foetus* [8, 57, 60–62].

Of the 19 pathogens examined in this study, all enteropathogens had zoonotic potential, except FECV, FPV, FIV, and *C. cayetanensis*, and their combined prevalence was 63.1%. Zoonotic enteropathogens can be transmitted through animal fecal matter and direct contact with the animal [63]. Thus, immunosuppression in the owners or veterinary staff could increase the risk of infection; information regarding the zoonotic potential of these organisms should be provided to owners or veterinary staff. Emerging zoonotic enteropathogens, which are a problem in humans, include *Salmonella* spp., *Campylobacter* spp., *C. coli,* and *C. jejuni,* with the latter two being the most common in human acute gastrointestinal disease [21, 64]. The prevalence of *Salmonella* spp. infection in cats with diarrhea was very low (0.25%) in this study, but has been reported to be as high as 6% in previous studies [1]. Although the prevalence of *Salmonella* spp. in this study was low, some cases have shown transmission from cats to humans [65, 66]. Similarly, a pet cat has also been reported as a source of transmission of EHEC infection to its owner [67]. In addition, because it is an important zoonotic pathogen, the fact that *Salmonella* spp. and EHEC were identified in even a small percentage of cat feces is important in terms of public hygiene; this co-infection should be carefully monitored.

There are some limitations to this study. First, it was difficult to conclude that the pathogens detected in this study were the cause of feline diarrhea. Some pathogens found in this study are also detected in healthy cats [1, 4]. Our study did not compare the results between diarrheic and asymptomatic cats. In addition, because this study was conducted using only stool samples and clinical information from patients from various animal hospitals, it is unclear whether the actual cause of diarrhea was the primary infection. Second, it was not possible to confirm whether antibiotics were used immediately before the test. The use of antibiotics might have influenced the results of this study. Third, this study was conducted retrospectively by extracting the results of a test commissioned by a commercial laboratory. Therefore, fecal collection methods may not have been consistent or followed properly, resulting in potential cross-contamination. However, because the laboratory specified the collection method in advance, the possibility of cross-contamination was very low. Finally, in this study, the detection of enteropathogens was performed only by real-time PCR. This method shows the highest sensitivity among laboratory tests, including enzyme-linked immunosorbent assays, bacterial culture, and virus isolation. However, it can lead to false-positive results because of the ability to detect pathogens in carriers [18, 68]. It is not possible to determine whether a specific organism is the cause of diarrhea based only on real-time PCR. To minimize this possibility, we considered the pathogen to be the causative agent of diarrhea only when the C_T_ value was less than 40.

## Conclusion

This study suggests that determining the etiology and clinical features of feline infectious diarrhea will facilitate decision making concerning treatments for infectious diarrhea and will help to prevent the spread of infection. Further studies and continuous monitoring are necessary to establish clinical information on various feline enteropathogens in diarrheic cats.

## Data Availability

The datasets analysed and the materials used for this study are available from the corresponding author upon reasonable request.

## Abbreviations

FECV: Feline enteric coronavirus
PCR: Polymerase chain reaction
FPV: Feline parvovirus
EPEC: Enteropathogenic *E. coli*
ETEC: Enterotoxigenic *E. coli*
EHEC: Enterohemorrhagic *E. coli*
RT: Reverse transcription
EIEC: Enteroinvasive *E. coli*
FIV: Feline immunodeficiency virus
